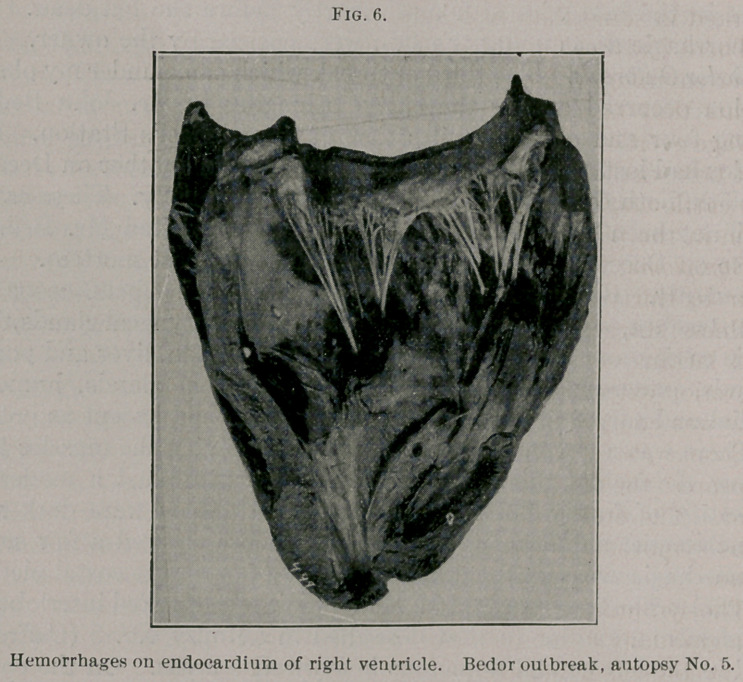# Hemorrhagic Septicæmia

**Published:** 1903-01

**Authors:** M. H. Reynolds

**Affiliations:** University of Minnesota, St. Anthony’s Park, Minn.


### HEMORRHAGIC SEPTICÆMIA.

By M. H. Reynolds, M D., V.M.,
UNIVERSITY OF MINNESOTA, ST. ANTHONY'S PARK, MINN.

Whether it is proper to speak of this as a disease or as a collection of diseases due to identical or similar germs may possibly be questioned, but, for the present at least, and until we have more light on the subject, we may speak of it all as hemorrhagic septicæmia. This disease is very interesting on account of several peculiarities. It is interesting because of its protean form; it is interesting because medical treatment, so far as we know, is absolutely useless and hopeless. We are utterly helpless in the matter of prevention because we have practically no information as to the method of infection or method of spread. Those who have had a chance to study the various outbreaks in Minnesota have been quite unable to trace any connection between one outbreak and another, or to trace a previous history for any given outbreak. It is proving interesting to those members of the veterinary profession who have been so fortunate or unfortunate as to come into contact with it, because of the extreme difficulty of making ante-mortem diagnoses. In some outbreaks that have been carefully studied, ante-mortem diagnosis for the first case at least was apparently impossible; but, on the other hand, there have always been plenty of opportunity for examinations post mortem, and here the evidence is usually clear.

There is a very practical side to this disease, especially when considered from the farmer's standpoint. It appears suddenly and under all sorts of conditions; a number of animals, usually a large proportion, die, and the disease disappears as suddenly as it came. The owner has lost a certain amount of property in live stock, without a trace of information as to whence the disease came or how soon the visit may be repeated. The owner would be especially interested in knowing the method of infection and the possible agencies through which his herd may receive a reinfection. If hemorrhagic septicæmia, like glanders, comes by a specially introduced infection, then he has a certain proposition to face. If, on the other hand, the micro-organism of hemorrhagic septicæmia, which resembles in all laboratory peculiarities the germs of swine-plague, are commonly present in less virulent forms or present under conditions where there is but limited opportunity for their development and the production of disease, then the farmer may expect an outbreak of it at any time and, so far as he knows, under any conditions, and he has no means of guarding against it—a quite different proposition.

*Etiology.* The specific cause of this disease is apparently bacillus bovisepticus, which cannot be distinguished from the bacillus of swine-plague by cultural or morphological characteristics. How this micro-organism spreads or how it gains entrance into the animal body is not known, but at present we suppose that the entrance may be affected by inoculation through the respiratory or the alimentary mucous membrane.

*History and Development.* The onset is usually sudden and most unexpected, and yet in some recent outbreaks of disease in which the presence of the micro-organism was demonstrated, the onset was quite slow and the cases were distinctly chronic. Hemorrhagic septicæmia is probably more prevalent than is generally supposed, and it is undoubtedly true that a great many outbreaks of this disease have been incorrectly diagnosed as anthrax, symptomatic anthrax, infectious cerebrospinal meningitis, cornstalk disease, and very possible as parturient paresis. From reports that have appeared in the veterinary journals at various times it is very evident to those who have had opportunity to study this disease that outbreaks of hemorrhagic septicæmia have appeared in a great many different sections of the United States at least, and have been incorrectly diagnosed. Cases which have been described in the East as cerebrospinal meningitis have been very plainly hemorrhagic septicæmia, and this is also true of cornstalk disease in the West.

Season and climatic conditions apparently have nothing to do with the prevalence, virulence, or disappearance of this disease. The mortality for the past few years during which it has been studied in Minnesota has been extremely high, the cases all ending abruptly in death, with the exception of a few outbreaks where the cases were chronic. These tended to disturb our supposed information concerning the disease, particularly in reference to its rapid and invariable fatality.

More recently an outbreak appeared under the observation of the writer where all cases gave uniformly clear ante-mortem symptoms of cerebrospinal meningitis, and yet examinations post mortem revealed in addition to the expected lesions of cerebrospinal meningitis the characteristic hemorrhages of hemorrhagic septicæmia, and the organism which is supposed to be the specific cause of the disease was demonstrated beyond reasonable question. (See "University Experiment Farm Outbreak," provisional report of Dr. Wesbrook.) In this outbreak, as in all the earlier ones, the mortality was very high; nine animals sickened and nine died.

*Symptoms.* The writer has had the privilege of studying closely the development and full history of about twelve cases. The temperatures were uniformly normal and subnormal, except in two cases, where the temperature rose rapidly just before death. There was nothing in the nervous disturbances that was especially diagnostic, except that in several cases the skin has been hypersensitive. The subjects have usually been disinclined to move about, apparently because movement caused pain. In an outbreak which occurred at the University Experimental Farm, and which came under the writer's daily observation, the prominent symptoms in all cases were those of cerebrospinal meningitis, but it would be very misleading to suggest that these nervous disturbances are characteristic of hemorrhagic septicæmia. Local lesions which correspond to the tumors of anthrax and symptomatic anthrax are very limited or wanting. The urine in many cases has been scanty or blood-stained, and this is also true of the bowel discharges. The examinations post mortem are very much more definite and satisfactory. The blood is apparently normal. Subcutaneous hemorrhages are common and vary greatly in size and intensity; in some cases they are large and the hemorrhagic condition is marked. In other cases the hemorrhages are punctiform, scattered, and few in number. The hemorrhages may appear almost anywhere in the subcutaneous tissues or involve any of the viscera. The spleen is not enlarged, but there may be hemorrhages on the surface. The hemorrhages usually have very sharply-defined borders and are easily recognized as hemorrhages. The serous membranes frequently show small hemorrhagic areas, and the heart, especially the auricles, are often intensely hemorrhagic.

We may summarize the symptoms as follows: As a rule the disease disappears suddenly; the case develops very rapidly and terminates fatally. The ante-mortem symptoms are very unsatisfactory from a diagnostic standpoint. The post-mortem symptoms are definite and as a rule easily recognized, and consist of more or less extensive hemorrhages which are sharply defined when they appear upon the surfaces of the viscera and serous membranes.

*Losses in Minnesota.* It is now about two years since this disease was recognized in Minnesota by Dr. Wilson of our State Board of Health Bacteriological Laboratory. During these two years there have been reported to the State Board of Health eighty outbreaks among Minnesota cattle; these outbreaks involved fifty-two different farms, appeared in twenty different counties, and resulted in the loss of 551 cattle..

It is safe to assume that a considerable number of other outbreaks appeared but were not reported.

Four outbreaks have come under the writer's personal observation, and in three of these the opportunity was unusually good for careful study of the cases from a clinical standpoint.

*Outbreaks Which Came Under Direct Observation.*

*Johnson Outbreak.* Dr. Wilson and the writer went to North Branch, Chisago County, October 30th, for the purpose of investigating a very virulent disease that had appeared in a certain herd. The previous history was rather unsatisfactory because indefinite and incomplete. It was learned that five calves belonging to another party had died in a certain pasture earlier in the season, probably in June or July. The history of these cases agreed with the recent cases which we went to investigate, in that the calves had died suddenly; there had been some slight local, diffused swellings, and, on skinning, dark red areas were noticed by the owner. Another neighbor had lost suddenly a cow some months before with symptoms and history that agreed closely with those of the present outbreak and the five calves previously mentioned.

Mr. Johnson reported that he had twenty cattle in his herd when the disease first appeared on September 15th. A portion of the pasture was dry; another portion quite low and wet, but without timber. None of the cattle has had access to standing cornstalks. He had lost two animals about November 1, 1899, from what he supposed was the same disease. These were calves about six months of age. The owner noticed that in these previous cases the manure was coated with blood or showed bloody streaks. During 1900 one was taken sick and died suddenly in July, another about the middle of October. He had lost also one roan steer calf, seven months old, which died some time early Monday morning, October 29th, and a red heifer calf about the same age, which died on the same Monday. Both deaths occurred suddenly. The former was noticed to limp some in walking, the trouble being apparently in the left front limb. These two calves were examined post-mortem by Dr. Wilson and the writer on Wednesday, October 31st.

The following parts were examined and all parts were normal except as noted. Parts examined: subcutaneous tissues, mucous membranes, heart, lungs and pleuræ, alimentary tract, bladder, post-pharyngeal, mediastinal, bronchial, mesenteric, portal, and inguinal lymphatic glands, kidneys, and spinal cord in the anterior cervical region.

*Autopsy* 1. A red steer calf in fair condition and about four months of age, had died about thirty-six hours prior to our visit. The carcass was moderately bloated, otherwise in fair condition for examination. The skin was discolored in places, especially where denuded of hair. The subcutaneous areolar tissues were emphysematous with fairly well-defined hemorrhages, especially marked at the throat and the adjacent portions of the inferior cervical region. The superficial muscles beneath these infiltrated areas were similarly involved. The surfaces of the limbs below the knees and hocks did not show hemorrhages, as in the cases previously reported by Drs. Wilson and Brimhall. There were no wounds of the skin near the feet that could be detected. Tracheal, œsophageal, and laryngeal mucous membranes show marked inflammation, being dark, swollen, and wet.

The kidneys were probably normal at the time of death, but when examined they were soft and showed numerous light yellowish areas about 8 mm. in diameter. The lungs showed a few small, sharply-defined, hepatized areas, markedly resembling the peculiar lesions of swine-plague. The owner had noticed that this calf was quite lame while sick, and it is interesting to note that in the examination one of the peculiar areas of the hemorrhagic septicæmia was found involving the shoulder muscles. Several articulations showed

FIG. 1.

Ulcerations of tibio-tarsal cartilage. Tibia and os calcis. Johnson outbreak, autopsy No. 1.

ulcerations of the articular cartilages, especially the humero-radial and tibio-tarsal. These ulcerations were about 31 mm. long by 12 mm. wide.

*Autopsy* 2. This was a red heifer calf, seven or eight months of age, in fair condition. The animal had been dead about forty-eight hours, but showed less post-mortem change than No. 1. So far as superficial parts are concerned, the post-mortem findings of No. 1 will apply very closely. This is also true of the lymphatic glands, mucous membranes and kidneys. The dura mater in the anterior cervical region had evidently been the seat of a very active inflammation. The lungs showed the peculiar hepatized areas of swine-plague, closely resembling those found in No. 1.

*Caffrey Outbreak*. The second outbreak studied by the writer in part with Dr. Wilson occurred near Cokato. My first information came in the following letter:

COKATO, November 26, 1900.

DR. M. H. REYNOLDS,

St. Anthony's Park, Minn.

DEAR DOCTOR: Mr. Caffrey, living two miles north, lost two cows about three days ago, and a third one is nearly dead now.

The first cow was apparently well at night, and was found dead in the morning. The next day another cow was taken sick and I was called. The second cow was comatose, uanble to rise, and died in about twenty-four hours.

*Symptoms.* This cow was lying on her breast with the head turned to one side, as in parturient paresis. She was comatose, her respiration stertorous, temperature 101°; pulse could not be taken.

*Post Mortem.* There was cherry-wine colored serum in the abdominal cavity; the small intestines were badly inflamed; the liver was slightly swollen, dark, and easily torn; the spleen was normal in size and a little darker in color than normal. The cephalic lobes of the lungs were inflamed, the heart had a parboiled appearance, and everything indicated a general septicæmia. Both lungs were congested.

This farm is situated at the southwest corner of Cokato Lake. Some of the land is low, some hilly.

These cattle were fed on cornstalks, hay, and shorts.

Yours respectfully,

H. A. HELA, M.D.C.

In company with Dr. Wilson, the writer reached Mr. Caffrey's place on November 29th. We learned that the Caffrey cattle had been kept in pasture as long as the grass was good, and were stabled at night. Later in the season they had been fed on wild hay, cornstalks, and shorts, the feed being apparently all fresh and good. The pasture in which the cattle had been during the summer and fall contained both high and low ground with some timber and brush. The owner had noticed in those cases which had died before we reached the place that the head had been drawn far back in some instances, and in some others the head was held in the flank as in parturient paresis, these positions being assumed shortly before death. He had noticed no superficial swellings, but said that the animals seemed to have irregular chills. He had also found blood-stained areas in all cases on the surface of the bodies after skinning. The sick animals had shown complete loss of appetite from the time they were first noticed ill. There had been no swine disease or chicken cholera in the neighborhood during the past season.

The following deaths, 1 to 6, inclusive, were reported to us by the owner:

*Death* 1. A six-year-old cow in fair condition had appeared normal in all respects on the evening of November 22d, and was found dead the next morning, the owner not having supposed that the cow was sick. The cow had died in an easy, natural position, as though resting. Evidently there had been no struggle.

*Death* 2. Another six-year-old cow was found down on November 22d, early in the morning; seven o'clock. She was unable to rise, held head in the flank, and died on the evening of the 23d. There was persistent constipation; no superficial tumors.

*Death* 3. A spring steer calf, in good condition, was first noticed sick on the evening of November 25th. This animal tried repeatedly to rise and failed. Died about 10 A.M., November 26th; constipation persistent.

*Death* 4. This was a four-months-old calf. The owner heard a noise in the stable about 2 A.M. on November 27th. He went out

FIG. 2.

General view of the small intestines. Caffrey outbreak, death No. 6, showing many small, sharply-defined hemorrhages on peritoneal surface.

to investigate and found the calf jumping into the manger and shoving the head against the wall. This calf died at 11 A.M., with the head drawn far back.

*Death* 5. This was a spring calf, supposed to be in perfect health until the morning of November 27th, when it was found almost dead. The animal died a little later. Constipation persistent.

*Death* 6. An eight-year-old black cow, in good condition, was first noticed sick November 28th, at 7 A.M., and found dead about 10.30 A.M. She dropped suddenly at 7.30 and subsequently tried repeatedly to rise, but could not. She lay on the left side with the head in the flank much of the time. Later the cow succeeded in getting on her feet, but fell suddenly with the posterior limbs spread outward and backward, the body dropping suddenly from a standing position to the ground. Later the cow drew up her limbs and lay over on one side in a rather natural position with the head swung backward. She struggled considerably, but later died easily and slowly.

*Autopsy.* The tissue lesions were rather severe. Both lungs showed considerable interlobular emphysema, which was marked in the ventral lobes. Petechiæ were especially marked on the caudal lobes. The right caudal lymphatic gland was dark, swollen, and showed petechiæ.

The diaphragm showed scattering petechiæ on the peritoneal surface of the tendon. The heart was markedly hemorrhagic, the hemorrhages being both superficial and deep.

The gall-bladder was filled with a dark bloody fluid. Its walls were infiltrated and œdematous and the surrounding tissues were œdematous.

FIG. 3.

Section of small intestine. Caffrey outbreak, death No. 6. Typical hemorrhages on peritoneal surface.

There were petechiæ on the third stomach penetrating the walls. Eighteen inches below the pylorus the duodenum was œdematous and bedded in a yellowish gelatinous mass. The small intestines showed well-marked petechiæ throughout. These areas were large and abundant.

There were petechiæ in the kidney substance and upon the surfaces. The bladder walls were hemorrhagic, the mucous membrane being much inflamed, thickened, and softened. There was a small quantity of bloody fluid in the bladder. Spleen petechiæ were abundant, and especially conspicuous on the inferior extremity.

*Death* 7. This was a black and white heifer in fair condition, seven months old. (See Fig. 4). This animal was first seen by Drs.

Wilson and Reynolds at 2 P.M., November 29th. The temperature was then 98.6°, the calf being out of doors on a very chilly day. The respirations were very shallow but normal in frequency. The pulse was not taken. This calf stood with the back arched, shivering, and apparently ready to fall at any minute. The hair was rough; there was a slight filling at the inferior cervical region, and the eyes were sunken. The muzzle was dry.

At 4.45 P.M. the calf was still out of doors, temperature 100.9°, pulse 72, full, soft, and fairly strong; respiration 20. The heart and lung sounds were normal so far as could be determined by auscultation.

FIG. 4.

Yearling heifer. Caffrey outbreak, death No. 7, thirteen hours before death.

At 7.30 P.M. the temperature was 101.1°, respiration, pulse, etc., about as at 4.45. The calf was now put in the stable out of the wind, but the stable was cold.

At 10 P.M. she was lying on the left side with the head resting forward on the ground. The pulse was 54 and much weaker, temperature 99.9°, respiration slightly irregular and somewhat jerky. The skin and underlying tissues over the body seemed very sensitive under pressure. This was especially noticeable over the abdomen. The calf had evidently failed rapidly since 7 P.M. The head was jerking spasmodically and unconsciously, the spasms affecting especially the cervical muscles. The pupils were dilated, muzzle dry, and the neck seemed to be filling slightly at the throat.

At 3.30 A.M. the calf was dead, lying flat on the side in a rather natural and easy position. There were noted slight rigor mortis, moderate tympanitis, and somewhat blood-stained feces. The animal had died, as nearly as could be estimated, about 2 A.M. The respiration had been slightly stertorous from 7.30 P.M. to 10 P.M., after which the animal was not seen until found dead.

*Autopsy.* There was considerable serum in the abdominal cavity and a small quantity in the pleural cavity. Both lungs were somewhat congested but showed no petechiæ. The trachea contained an abundance of frothy material and the bronchi were moderately injected. The œsophageal mucous membrane was normal. The diaphragm had a few small hemorrhages on the pleural side. The liver showed a few small hemorrhages on the spigelian lobe. There were a few moderate hemorrhages on the heart surface and on the endocardium. The duodenum was in a condition very similar to that described in the post-mortem record of death No. 6. It was involved in a gelatinous mass filled with yellow serum about ten inches from the pylorus. The ileum was injected and the mucous coat showed areas of distinct inflammation. The rectal mucous membrane was very much inflamed. Subcutaneous hemorrhages were present, but small and not well marked. None were noticed on the inferior cervical region or on the lower portion of the limbs. The plainest and most typical hemorrhages were on the liver, as already noted. Both the parietal peritoneum and parietal pleura showed very little that was abnormal.

There was an old wound in the abdominal wall extending into the rumen about one inch in diameter. This injury must have occurred at more than a month prior to this examination. The stomach was adherent around the border of the abdominal wound, and the abdominal cavity was thus shut off.

The left humero-radial articulation showed one ulcer involving the articular cartilage about 25 mm. long, oval in shape. The left tarsal articulation showed an oval ulcer about 38 mm. long. The other articulations appeared normal.

Post-pharyngeal glands were enlarged, dark, and markedly hemorrhagic. The dura mater was moderately congested, with a little serum in the canal at the atlo-axoid articulation. The bladder was normal and very much distended with normal looking urine.

The tissue lesions in this case were neither extensive nor severe, evidently a case of toxine poisoning.

*Death* 8. A red heifer, about eighteen months old and in good condition, was first noticed sick November 30th at 7 A.M. She had previously been in good health, so far as known. When first noticed she was bellowing occasionally and standing apart from the other cattle. She refused her morning feed and was put in the stable about half an hour later. This calf soon went down and did not rise.

At 7.30 the temperature was 99°, respiration 22, pulse 14 and good. Respirations were full but somewhat stertorous. The horns were cold. Evidently the circulation was poor. Light-colored feces were passed.

At 10.30 A.M. the temperature was 97.8°, having fallen 1.2 degrees during the previous three hours. Respirations were now 24 and markedly stertorous. Pulse could not be counted. The subject was failing rapidly. This case also showed the hypersensitive condition of the skin.

At 12 M. the temperature was 97.8°, pulse was feeble and could not be counted. The respirations were still stertorous, the expiration being accompanied by spasmodic jerking of the abdominal muscles.

At 2.30 P.M. the temperature was 97°, respiration about 24, and the heifer was lying stretched on one side.

At 4 P.M. the temperature was 96°, respirations 24; pulse could not be counted. The heifer was groaning with each expiration. The head was very much drawn back and the body still sensitive under pressure. The animal died at 10.30 P.M.

*Autopsy.* A hasty post mortem by the owner discovered what he described as bruised areas under the skin.

*Death* 9. A spotted heifer, three years old, was noticed sick on December 2d, at 4 P.M. She died at about 5 P.M., having apparently been in the best of health until shortly before she fell dead. No hemorrhagic areas under the skin were noticed by the owner.

*Bedor Outbreak.* The third outbreak which came under my observation occurred among the cattle belonging to Mr. John Bedor, living four and one-half miles east of St. Michael's Station. Mr. Bedor had lost one animal on December 1st and another on December 5th, both dying very suddenly and unexpectedly. These cattle had not been standing in corn. The writer visited Mr. Bedor's place on December 7th, and held examination post mortem.

*Autopsy.* Bedor death No. 2. Parts examined: subcutaneous tissues, trachea, œsophagus, dura and cord, post-pharyngeal glands, thoracic cavity and contents, alimentary tract, spleen, liver and portal glands, pancreas, bladder, peritoneum, inguinal glands, humero-radial and carpal articulations. All parts normal except as noted.

There was a circular hemorrhagic area involving the muscles just below the ischial tuberosity. The trachea contained a moderate quantity of frothy fluid. Post-pharyngeal glands were dark and hemorrhagic, but normal in size. The pleura showed a few small hemorrhagic areas on the diaphragm and a few on the costal pleura. (Fig 5.) The lungs showed in one cephalic lobe marked interlobular emphysema, similar to that described in autopsy No. 3 (Caffrey), and in the left caudal numerous hemorrhagic areas. On the endocardium of the right ventricle of the heart were several fairly well-marked hemorrhages. (Fig. 6.) Bronchial and mediastinal glands were not carefully examined, but were probably normal. There was one circular hemorrhagic area on the third stomach, quite typical. There were a few typical hemorrhages, 5 to 10 mm. in diameter, on the capsule of the liver. The duodenum and rectal mucous membranes were markedly inflamed and swollen.

FIG. 5.

Hemorrhages on costal pleura. Bedor outbreak, autopsy No. 5, death No. 2.

FIG. 6.

Hemorrhages on endocardium of right ventricle. Bedor outbreak, autopsy No. 5.

Mr. Ralph Richner, a near neighbor to Mr. Bedor, reported that he had approximately twenty head of cattle in his herd November 20th. Mr. Richner lost nine, most of the animals dying very suddenly, and the entire nine within a few days after the first case, which appeared on November 20th. His cattle had been fed shocked corn and other dry feed in the yard, and had not been standing in cornstalks at all. On skinning the animals and opening the carcasses the owner noticed that the livers and stomachs were spotted. The intestines were not especially noticed. He would probably not have noticed any petechiæ on the intestines, even had they been present. Dark bloody spots were noted under the skin in some cases.

*University Experiment Farm Outbreak. History.* On June 6th, seven cows, which had given a normal flow of milk in the morning, gave practically none in the evening. Otherwise the cows were apparently normal.

These cows were all noticed to be slightly ailing the next morning, with the exception of Dell 2. This cow was down and could not be gotten up. The others showed little except dulness. There was no rise of temperature; no evidence of pain or discomfort. When they attempted to walk the gait was more or less irregular, resembling very much the gait of milk fever in its early stage. This became true of all the cases sooner or later, and was of course more marked in some than in others.

There had been nothing new or unusual in the care or feed or other environments of these cattle, except that for a few days and nights they had been kept in a pasture which had received some sewage overflow from our filter beds, by reason of recent rains. A salt box was located near the point where this overflow stood and the cattle unquestionably drank of this water. No other cattle had been in the pasture for ten days.

*Symptoms; First Period.* The symptoms during the first twenty-four to thirty-six hours were not marked except as to continued dullness, staggering gait, and cold extremities. The skin was harsh and lacking sensation. This loss of skin sensation began at the posterior extremities and gradually extended forward. The milk flow was completely checked, or practically so in all cases.

*Second Period.* After twenty-four to thirty-six hours diarrhœa appeared, the discharge being dark and thin with a very disagreeable odor. The breath in some cases was noticed to be offensive. Nervous symptoms gradually developed and were very uniform in all cases.

The symptoms during the second period were those which belong to a gradually developing nervous disturbance and were very typical of cerebrospinal meningitis. The inability to walk naturally was continued, the gait being irregular and weak. The neck was usually bent to one side and the muscles, particularly of the face and neck, were spasmodically contracted. During this period the animals, with the exception of Countess, a large Holstein cow, were still quiet, moving around very little; but the eyes showed a wild, unnatural expression. The skin continued to lose sensation progressively forward. Countess was continually groaning, or rather grunting, with each respiration, but not in evident pain. During this second period she commenced to chew in a nervous and very persistent manner, with more or less profuse flow of saliva. It is also to be noted that the temperatures remained normal or subnormal during this period.

*Third Period.* This was one of intense activity. The eyes continued to grow more wild and unnatural, the grinding of the jaws more active and constant; the convulsion of the face and neck muscles became more intense, and then gradually a period of intense restlessness and activity, and death ended the scene in every case.

*Post-mortem Symptoms.* Several of these animals were examined and the symptoms as seen on examination post mortem were fairly uniform.

Meningitis involving the spinal cord or brain or both these organs was invariably present. In addition to this there were hemorrhages involving the subcutaneous tissues and lymphatic glands in various portions of the body; also the pleuræ, pericardium, and surfaces of various internal organs, particularly the lungs and auricles of the heart.

Evidently we had here meningitis; not the specific form of the disease, but one probably due to another germ. The lesions seen on post mortem are very suggestive of a hemorrhagic septicæmia infection.

*Diagnosis.* The veterinarians present, Drs. Lyford, Brimhall, Annand, and Reynolds, agreed that the clinical symptoms and the results of examination post mortem warranted a diagnosis of cerebrospinal meningitis; but the hemorrhagic conditions made it evident that we did not have the recognized specific type of the disease to deal with. (See *Provisional Report on Bacteriological Examination*, etc., by Dr. Wesbrook.)

*Source of Infection.* Owing to the fact that this particular outbreak occurred in a certain small portion of our herd and did not spread to other cattle on the farm, we were at first inclined to suspect the water in one of our pastures. The affected lot of cattle (our milking dairy cows) had been recently turned into this pasture and a certain small pond had been contaminated by sewage overflow from our filter bed, as already noted. But the fact that a few days later a virulent case of the same disease appeared in a heifer which had not, so far as known, had access to this water, but had been kept in an adjoining pasture, seemed to weaken this theory. In addition to this, the further fact that an experimental cow which was given this water only for a period of two weeks gave her normal flow of milk and remained in perfect health, seems to disprove the sewage-water theory as to the source of infection. A careful survey of the history and surrounding conditions leaves us still in the dark except as to the following incident: A sheep died about a year before of typical hemorrhagic septicæmia. It is possible that the infection came remotely from this sheep and that the meningitis was due to germ infection, the germ of hemorrhagic septicæmia being the exciting cause. It should be shown in further explanation that the sheep in question was buried in a field remote, considerably more than a quarter of a mile from the pastures wherein the disease appeared among cattle, although drainage is from this field toward the pasture in question. Other cattle have been kept during the interval in these pastures without harm. We do not know where the sheep received its infection. The cattle may have been infected from the same original source, or possibly there was an indirect infection from the dead sheep; but the latter theory seems very improbable. The sheep in question developed its disease and died in the sheep barn practically surrounded by other sheep, and yet we had no other cases among sheep at that time and none since.

June 19, 1902.

Dr. M. H. Reynolds,
St. Anthony's Park, Minn.

Dear Doctor Reynolds: Enclosed please find provisional report on the epizootic amongst the cattle at the State Experiment Station.

Yours very truly,
F. F. Wesbrook,
*Director.*

(To be continued.)

---

Dr. Austin Peters, of Massachusetts, in charge of the State veterinary control work, failed to find evidence of the existence of foot-and-mouth disease among the cattle coming into the State until it was prevalent in some four of the New England States.

Dr. A. F. Schreiber, of Philadelphia, Pa., chief meat inspector of the city, was seriously ill with pneumonia in November, following an attack of bronchitis.

Dr. Charles Ellis, of St. Louis, Mo., now fills the rôle of Veterinarian to the City Board of Health of that municipality.

## Figures and Tables

**Fig. 1. f1:**
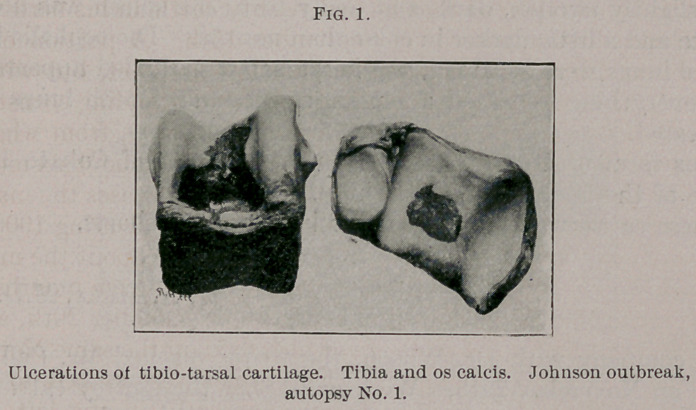


**Fig. 2. f2:**
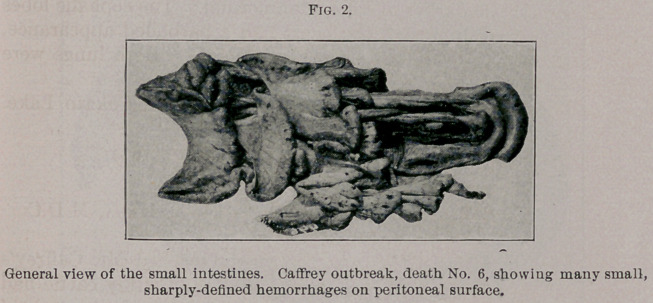


**Fig. 3. f3:**
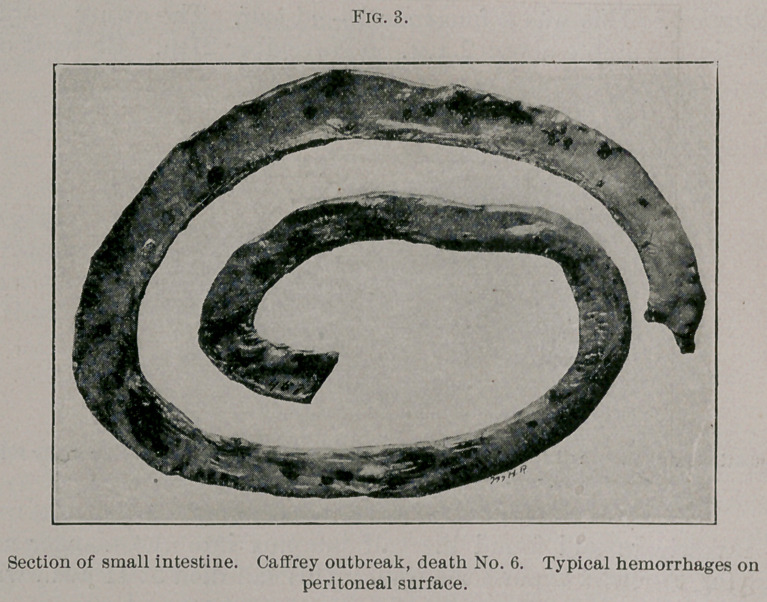


**Fig. 4. f4:**
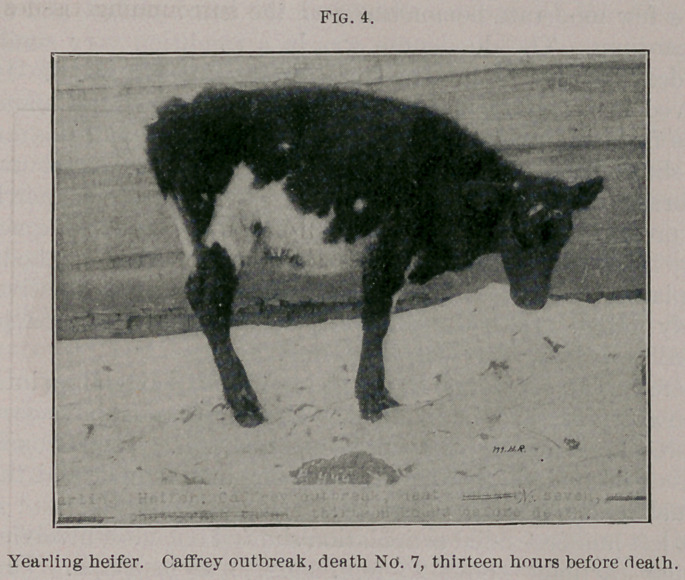


**Fig. 5. f5:**
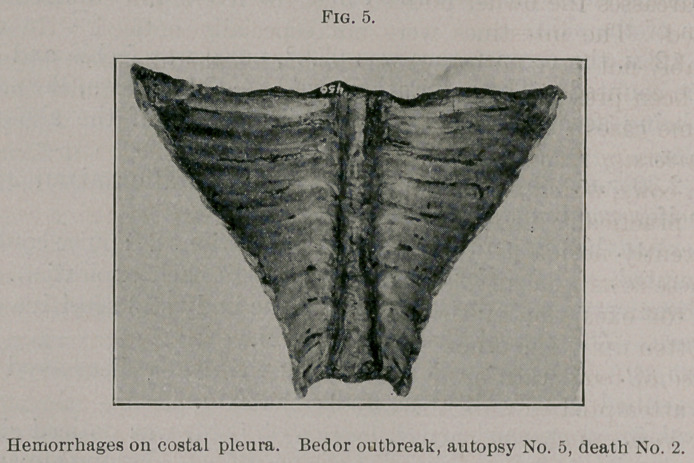


**Fig. 6. f6:**